# The Role of Neutrophils during Mild and Severe Influenza Virus Infections of Mice

**DOI:** 10.1371/journal.pone.0017618

**Published:** 2011-03-14

**Authors:** Michelle D. Tate, Lisa J. Ioannidis, Ben Croker, Lorena E. Brown, Andrew G. Brooks, Patrick C. Reading

**Affiliations:** 1 Department of Microbiology and Immunology, University of Melbourne, Melbourne, Victoria, Australia; 2 Walter and Eliza Hall Institute of Medical Research, Melbourne, Victoria, Australia; 3 WHO Collaborating Centre for Reference and Research on Influenza, North Melbourne, Victoria, Australia; Erasmus Medical Center, Netherlands

## Abstract

Neutrophils have been implicated in both protective and pathological responses following influenza virus infections. We have used mAb 1A8 (anti-Ly6G) to specifically deplete LyG6^high^ neutrophils and induce neutropenia in mice infected with virus strains known to differ in virulence. Mice were also treated with mAb RB6-8C5 (anti-Ly6C/G or anti-Gr-1), a mAb widely used to investigate the role of neutrophils in mice that has been shown to bind and deplete additional leukocyte subsets. Using mAb 1A8, we confirm the beneficial role of neutrophils in mice infected with virus strains of intermediate (HKx31; H3N2) or high (PR8; H1N1) virulence whereas treatment of mice infected with an avirulent strain (BJx109; H3N2) did not affect disease or virus replication. Treatment of BJx109-infected mice with mAb RB6-8C5 was, however, associated with significant weight loss and enhanced virus replication indicating that other Gr-1^+^ cells, not neutrophils, limit disease severity during mild influenza infections.

## Introduction

Neutrophils are recruited to the respiratory tract following influenza virus infections of humans and mice [1] and large numbers infiltrate the airways following infection of mice with highly pathogenic viruses such as the reconstructed 1918 H1N1 virus and strains of H5N1 [2], [3]. Current evidence suggests that neutrophils play a protective role following infection of mice with (i) human virus strains of intermediate virulence [4], [5], (ii) the mouse-adapted A/PR/8/34 strain (PR8, H1N1) [6], [7], [8], or (iii) a virulent recombinant influenza virus expressing the hemagglutinin (HA) and neuraminidase (NA) of a 1918 pandemic H1N1 virus [3]. However, depletion of neutrophil-attracting chemokine MIP-2 was associated with reduced neutrophil recruitment and a milder lung pathology following infection of mice with PR8, suggesting that dysregulated or excessive neutrophil responses in the airways may contribute to disease during severe influenza infections [9]. Therefore, the specific role of neutrophils during mild and severe influenza virus infections remains unclear.

Treatment of mice with mAb RB6-8C5 (anti-Ly6C/G or anti-Gr-1) has been widely used to deplete neutrophils in a range of murine models of infection and inflammation (reviewed by [10]), including influenza virus infection [3], [4], [8]. However, extending the findings of Daley *et al.* [11], we have demonstrated that mAb RB6-8C5 is not highly specific for neutrophils but binds a range of additional leukocyte populations in the lung of influenza virus-infected mice, including CD8^+^ T cells, macrophages, NK cells and conventional and plasmacytoid dendritic cells (cDC and pDC, respectively). Binding of RB6-8C5 to these cells correlated with expression of Ly6C, not Ly6G [5] making interpretation of studies using mAb RB6-8C5 to induce neutropenia complicated by the unwanted depletion of other Gr-1^+^ leukocytes. We showed that mAb 1A8 binds exclusively to Ly6G^high^ neutrophils in the airways of influenza virus-infected mice and specific depletion of Ly6G^+^ cells with mAb 1A8 lead to exacerbated disease in mice infected with virus strain HKx31 (H3N2).

In preliminary studies, we observed that virus strains of low (BJx109, H3N2), intermediate (HKx31, H3N2) or high virulence (PR8, H1N1) in mice differed markedly in their ability to recruit neutrophils to the airways. Given that neutrophils have been implicated in both protective and pathological responses following influenza virus infections, we investigated the role of neutrophils *in vivo* following infection with virus strains known to differ in virulence in mice. Moreover, given the widespread use of mAb RB6-8C5 to deplete neutrophils in mice [3], [4], [11], [12], [13], [14], [15], we compared the effects of treating mice with either mAb 1A8 or mAb RB6-8C5 to deplete Ly6G^+^ cells or Ly6C^+^/Ly6C^+^ (i.e. Gr-1^+^) cells, respectively.

## Results

### Influenza virus strains differ in their virulence for mice

**Figure 1:**
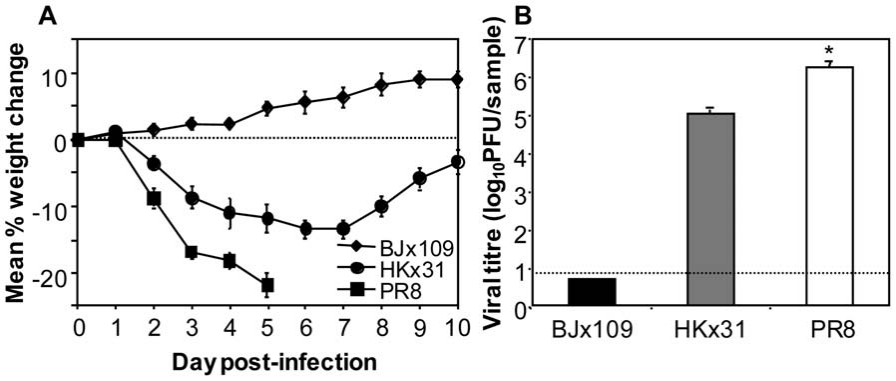
Virulence of influenza viruses for C57BL/6 mice. Mice were infected with 10^5^ PFU of BJx109, HKx31 or PR8. (A) Weight loss. Mice were weighed daily and results expressed as mean percent weight change of each group (± SEM), compared to the weight immediately prior to infection. (B) Titres of virus in the lungs at day 5 post-infection. Lungs were removed and titres of infectious virus were determined in clarified homogenates by standard plaque assay. Data represent mean virus titres ± 1 SEM. The detection limit for the assay is indicated by the dotted line. Data shown are for groups of 5 mice and are representative of 2 or more independent experiments. * = virus titres from PR8-infected mice were significantly higher than those from HKx31-infected mice (p<0.001, one-way ANOVA).

HKx31 (H3N2) and BJx109 (H3N2) are high-yielding reassortants of PR8 with A/Aichi/2/68 (H3N2) and A/Beijing/353/89 (H3N2), respectively, and bear H3N2 surface glycoproteins and internal components derived from PR8. We first compared the weight loss (Fig. 1A) and viral loads (Fig. 1B) of B6 mice following intranasal infection with 10^5^ PFU of PR8, HKx31 and BJx109. Mice infected with BJx109 did not lose weight nor did they display signs of clinical disease at any time during the 10-day monitoring period. HKx31-infected mice showed transient weight loss over the first week of infection but recovered thereafter and no animals succumbed to infection. In contrast, mice infected with PR8 lost weight rapidly, displayed evidence of severe clinical disease (huddling, laboured breathing; data not shown) and were euthanized 4–5 days post-infection. Therefore these virus strains were classified to be of low (BJx109), intermediate (HKx31) or high (PR8) virulence in the murine model of influenza infection.

Viral loads in the lungs of mice were determined 5 days after infection with each of the viruses (Fig. 1B). This time-point was chosen as it corresponded to the time at which PR8-infected mice succumbed to disease (Fig. 1A). BJx109 induces mild disease and is cleared from lungs by day 5 post-infection (Fig. 1B), although high virus titres can be recovered when components of innate immunity, such as airway macrophages, are depleted [16]. At day 5 post-infection, virus titres were markedly higher in the lungs of HKx31-infected mice and were 10–100-fold higher again in lungs of PR8-infected mice. Thus, a direct correlation was observed between virulence for mice and the ability of each virus strain to replicate in the airways (i.e. PR8>HKx31>BJx109).

### Neutrophil recruitment to respiratory tract following infection of mice with BJx109, HKx31 or PR8

**Figure 2:**
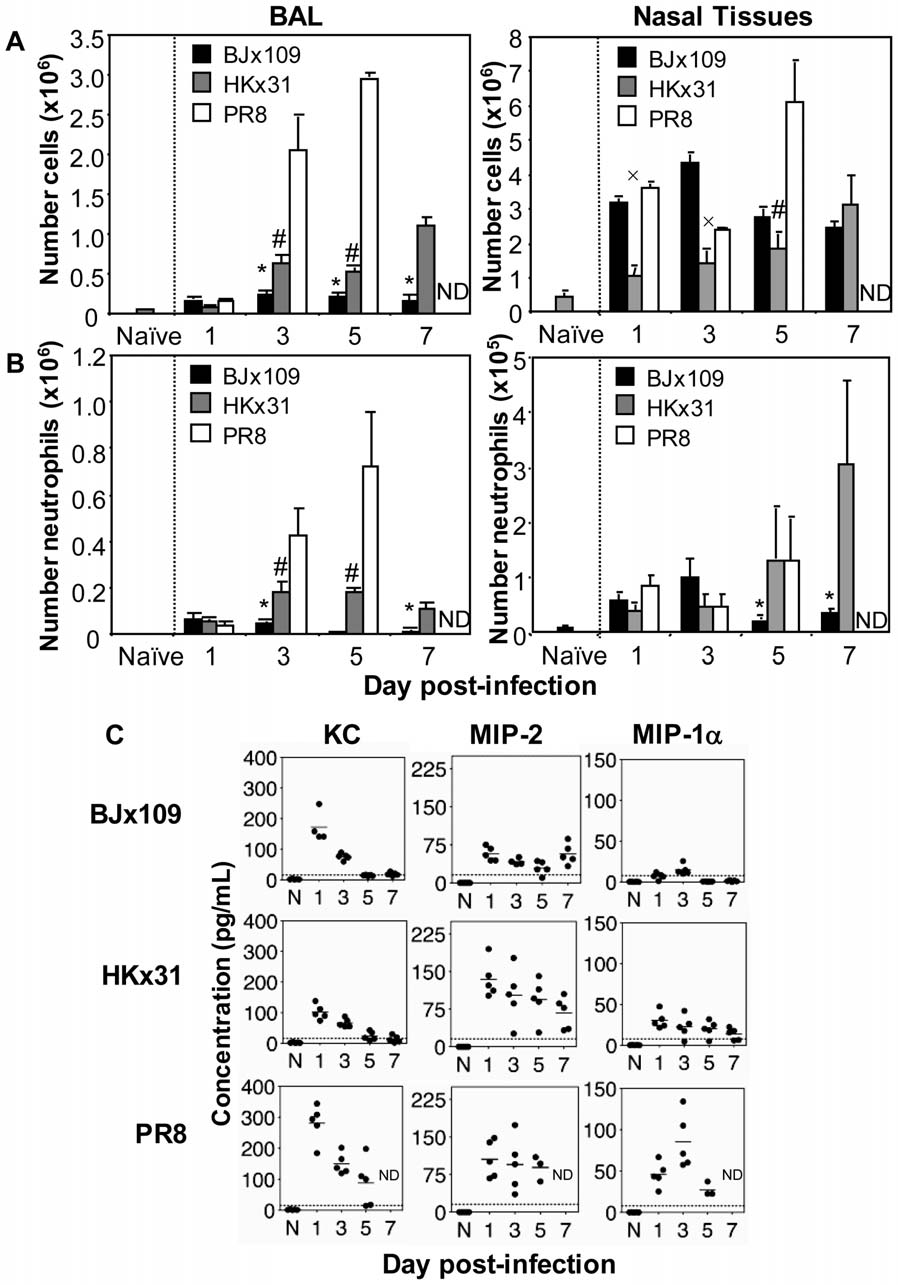
Neutrophil response in the airways after infection of mice with BJx109, HKx31 or PR8. Groups of 5 mice were infected with 10^5^ PFU of each virus via the intranasal route and at the times indicated, mice were killed and lavage performed. Numbers of (A) total BAL and nasal tissue cells, and (B) total BAL and nasal tissue neutrophils from virus-infected mice. Cell numbers in the BAL fluids from naïve mice are included for comparison. Neutrophils were identified by flow cytometry as CD45^+^ Gr-1^high^ cells. A minimum of 50,000 living cells (PI^−^) were collected and analyzed from each mouse. * = BJx109 is significantly reduced compared to HKx31 and PR8; ^#^ = HKx31 is significantly reduced compared to PR8; ^x^ = HKx31 is significantly reduced compared to BJx109 and PR8 (*p*<0.05, one-way ANOVA). (C) Levels of KC, MIP-2 and MIP-1α in BAL supernatants at various times after influenza virus infection. Naïve (N) animals were included for comparison. Chemokine levels were determined by ELISA and results are expressed in pg/mL. The detection limit for each ELISA is indicated by the dotted line. ND = not done as all PR8-infected mice were euthanized at day 5 post-infection.

The cellular inflammatory response to influenza virus infection was assessed in the lung (via analysis of bronchoalveolar lavage (BAL)) and in the upper respiratory tract (via analysis of collagenase-treated nasal tissues) at various time-points after infection with 10^5^ PFU of BJx109, HKx31 or PR8. First, viable counts were performed to determine total cell numbers recovered from BAL (Fig. 2A, left panel) or nasal tissues (Fig. 2A, right panel) of uninfected or virus-infected mice. Low numbers of BAL cells were recovered from BJx109-infected mice at days 1, 3, 5 and 7 post-infection (Fig. 2A, left panels). Infection of mice with an equivalent dose of HKx31 led to a greater accumulation of cells in the airways and by day 5 post-infection BAL cell numbers were ∼2.5-fold higher than those recovered from BJx109-infected mice. PR8 infection was associated with the progressive recruitment of inflammatory cells to the airways, such that by day 5 BAL cell numbers were ∼14-fold and ∼6-fold higher than cell numbers from BJx109- and HKx31-infected mice, respectively.

Cell numbers in the nasal tissues of mice infected with BJx109 peaked at day 3 post-infection (Fig. 2A, right panel), whereas those from HKx31-infected mice increased progressively from day 1 to day 7 post-infection. Note that while high total cell numbers were recovered from the nasal tissues of BJx109-infected mice at days 1 and 3 post-infection (Fig. 2A, right panel), neutrophil numbers were not significantly different between groups infected with each virus at these time points (Fig. 2B, right panel). Infection with PR8 induced recruitment of larger numbers of leukocytes to nasal tissues and by day 5 post-infection, cell numbers were ∼2-fold or ∼3-fold higher than numbers recovered from BJx109- or HKx31-infected mice, respectively.

In preliminary experiments, differential staining of cells recovered from the BAL and nasal tissues of virus-infected mice indicated that neutrophils were a prominent component of infiltrates during the early (days 1–3, BJx109 and HKx31) and later (day 5, PR8) stages of influenza infection (data not shown). Therefore, flow cytometry was used to quantify neutrophil numbers in BAL and nasal tissues at various times after infection with 10^5^ PFU of BJx109, HKx31 or PR8 (Fig. 2B). In previous studies, we have used cell sorting and morphological analysis to demonstrate that >95% of CD45^+^ Gr-1^high^ cells in BAL were neutrophils [4], [5]. The neutrophil response to BJx109 and HKx31 peaked 1–3 days after infection and numbers were markedly higher in BAL fluids from HKx31-infected mice at all time-points tested. The pattern of neutrophil recruitment into the BAL following inoculation with PR8 (Fig. 2B) was notably different as neutrophil numbers were low at day 1 post-infection, but increased progressively through to day 5 post-infection. Thus, during PR8 infection the accumulation of BAL cells, including neutrophils, correlated with the development of severe disease. Neutrophils were recruited into the nasal tissues following infection with each virus strain and peak neutrophil numbers in BJx109-infected mice were recovered 3 days after infection. In contrast, neutrophil numbers increased over time following infection with either HKx31 or PR8 and similar neutrophil numbers were recovered from nasal tissues 5 days after infection with either virus strain.

In general terms, the pattern of the neutrophil infiltration into the BAL following infection with BJx109, HKx31 or PR8 correlated with the expression of neutrophil-attracting chemokines KC, MIP-1α and MIP-2 in the airways (Fig. 2C). KC and MIP-2 were detected in BAL fluids from BJx109-infected mice 1 day after infection and remained low thereafter. HKx31 induced a similar pattern of chemokines, but with higher levels of MIP-2 and additional induction of low levels of MIP-1α. PR8 induced relatively high levels of each of the 3 chemokines, although levels were quite variable in BAL fluids at each time-point.

### Murine neutrophils are not susceptible to infection by influenza virus

Given the differing patterns of neutrophil recruitment to the airways following infection with BJx109, HKx31 or PR8 (Fig. 2B), we next investigated the susceptibility of purified murine neutrophils to infection by each virus strains *in vitro*. First, aliquots of 10^6^ purified bone marrow-derived neutrophils or murine airway epithelial cells (LA-4) were incubated with 10^7^ PFU of BJx109, HKx31 or PR8 for 1 hr at 37°C, washed to remove excess virus and following culture for an additional 2 or 24 hrs, supernatants were collected, clarified and the levels of infectious virus using a standard plaque assay on MDCK cells. Titres of infectious virus had increased ∼100-fold in supernatants from BJx109-, HKx31- and PR8-infected LA-4 cells between 2 and 24 hrs post-infection, consistent with productive infection of epithelial cells (data not shown). In contrast, titres of infectious virus did not increase between 2 and 24 hrs in supernatants from virus-exposed neutrophils, confirming that they do not support productive infection by BJx109, HKx31or PR8 (data not shown).

**Figure 3:**
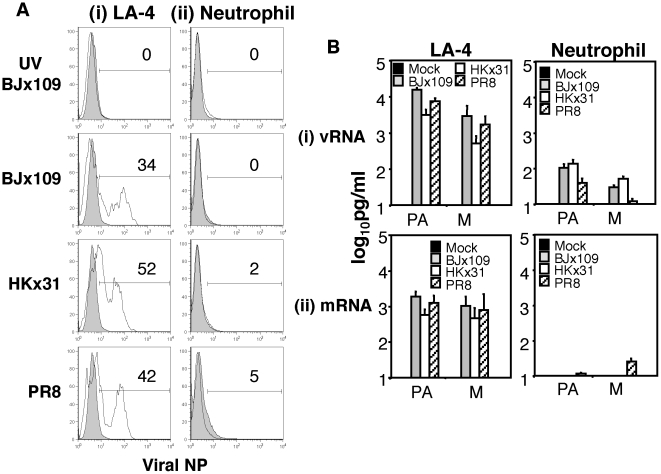
Neutrophils are not susceptible to influenza virus infection. Mature murine neutrophils were purified from the bone marrow as described in Materials and Methods. Aliquots of 10^6^ (i) LA-4 epithelial cells, or (ii) neutrophils were incubated with 10^7^ PFU of BJx109, HKx31 or PR8. Control cells were incubated with BJx109 that had been UV-inactivated to destroy virus infectivity (UV BJx109). After 1 hr, cells were washed and incubated an additional 8 hrs. (A) Cells were fixed and levels of intracellular viral NP protein determined by flow cytometry. Histograms shown represent cells incubated with virus (grey) or uninfected control cells (white). A gate was set to include 5% of cells in the uninfected control and the percentage of cells staining positive for intracellular NP are shown relative to this in each panel. (B) RNA was extracted from cells and levels of (i) vRNA and (ii) mRNA encoding NP, PA and M genes determined via quantitative RT-PCR. Cells incubated in media alone (mock) were included as a control. Data is expressed in pg/ml and represent the mean ± SD of 3 independent experiments.

Next, we incubated LA-4 cells and purified neutrophils with 10^7^ PFU of each virus strain (as described above) and, following washing, cells were cultured for a further 8 hrs. After this time, cells were fixed and stained for intracellular expression of newly synthesized viral NP. Note that cells incubated with UV-treated BJx109 (to inactivate infectious virus) were included as controls. LA-4 epithelial cells were infected by each virus strain (Fig. 3Ai), whereas viral NP was not detected in cells incubated with UV-inactivated BJx109. In contrast to LA-4 cells, viral NP protein was not detected following incubation of neutrophils with BJx109, HKx31 or PR8 (Fig. 3Aii), indicating that murine neutrophils are not susceptible to infection by BJx109, HKx31 or PR8 viruses.

To determine if neutrophils were susceptible to virus entry and/or the early stages of virus replication (via detection of vRNA) and/or translation (via detection of mRNA), we incubated LA-4 cells or purified neutrophils with influenza viruses (as described above) and, at 8 hrs post-infection, RNA was extracted and quantitative RT-PCR performed to determine levels of vRNA and mRNA for genes encoding PA and M (Fig. 3B). vRNA was not detected in mock-infected LA-4 cells, but high levels were detected in LA-4 cells incubated with either BJx109, HKx31 or PR8 (Fig. 3Bi, left panel). In contrast, vRNA was below detection or present at low levels in murine neutrophils incubated with virus (Fig. 3Bi, right panel). A similar pattern of viral mRNA expression was observed with high levels recorded in LA-4 cells incubated with BJx109, HKx31 or PR8 (Fig. 3Bii, left panel) but very low/undetectable levels recorded in neutrophils (Fig. 3Bii, right panel). Thus, while murine airway epithelial cells support genomic replication and productive infection by BJx109, HKx31 and PR8 viruses, murine neutrophils do not.

### Effect of treatment with mAb 1A8 or mAb RB6-8C5 on the virulence of BJx109, HKx31 and PR8 for mice

mAb RB6-8C5 (anti-Ly6C/G or anti-Gr-1) has been used extensively by researchers to deplete neutrophils from mice [3], [4], [11], [12], [13], [14], [15], however use of this mAb is complicated by its ability to bind to (and therefore deplete) additional leukocyte populations [4], [11], [17], [18], [19], [20]. We have previously utilized mAb 1A8 (anti-Ly6G) to specifically deplete neutrophils from the airways following infection of mice with a low dose (10^2^ PFU) of HKx31 [5]. While cell sorting confirmed that Gr-1^high^ cells may be classified as neutrophils [5], we demonstrated that a range of additional leukocyte subsets recovered from the lungs of HKx31-infected mice, including CD8^+^ cells, NK1.1^+^ cells, NKT cell, macrophage, cDC and pDCs, expressed intermediate levels of Gr-1 and are therefore likely to be depleted following treatment of mice with anti-Gr-1 antibodies [5]. In contrast, mAb 1A8 bound exclusively to Ly6G^high^ neutrophils in the airways of virus-infected mice. Moreover, BJx109, HKx31 and PR8 showed marked differences in their ability to induce leukocytes, including NK cells and CD8 T cells [21] to the airways, further complicating the use of mAb RB6-8C5 to compare the role of neutrophils following infection of mice with strains of different virulence.

To examine the role of neutrophils during infection with BJx109, HKx31 and PR8, mice were treated with mAb 1A8 prior to and during infection with 10^5^ PFU of virus. Briefly, mice were treated with purified antibodies (0.5 mg i.p. and 0.2 mg i.n.) one day prior to infection and every second day thereafter, as described in Materials and Methods. Control mice received an equivalent treatment of purified rat IgG and mice were also treated with mAb RB6-8C5 for comparison. Mice were monitored daily for signs of disease, changes in body weight and survival. Effective neutrophil depletion (>90%) was confirmed by morphological analysis of blood and BAL cells (data not shown). Uninfected mice receiving a similar regime of purified mAb 1A8, RB6-8C5 or rat IgG did not lose weight or display any physiological abnormalities over a 10-day monitoring period (data not shown).

**Figure 4:**
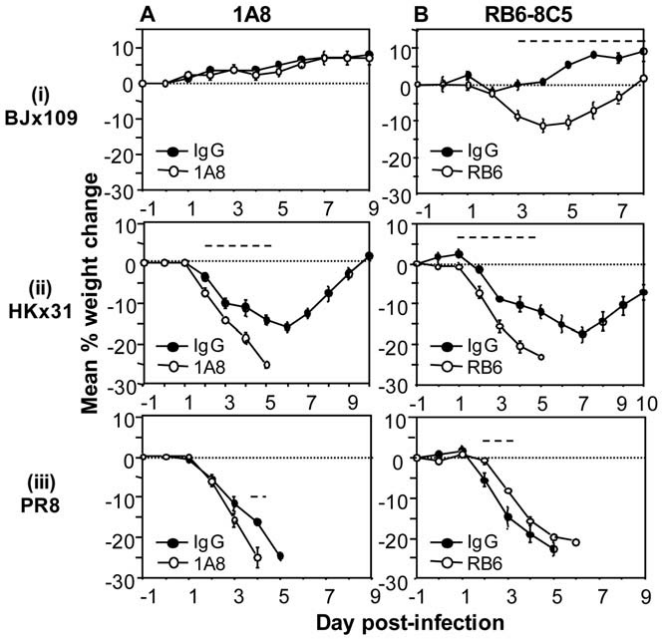
Effect of mAb 1A8 and mAb RB6-8C5 treatment of mice prior to and during infection with BJx109, HKx31 or PR8. Groups of 5 B6 mice were treated with purified (A) anti-Ly6G (1A8) or (B) anti-Gr-1 (RB6-8C5) antibodies 24 hrs prior to virus infection and every second day thereafter. Control mice received rat IgG (IgG). Data show weight loss for mice infected with 10^5^ PFU of (i) BJx109, (ii) HKx31 or (iii) PR8. Mice were weighed daily and results expressed as the mean percent weight change of each group (± SEM), compared to original body weight. Animals that had lost ≥25% of their original body weight and/or presented with evidence of pneumonia were euthanized. Data shown are from one experiment and are representative of two independent experiments. The dashed line indicates days on which the weight loss induced by treatment with (A) mAb 1A8 or (B) mAb RB6-8C5 was statistically different to IgG-treated controls (*p*<0.05, Student's *t*-test).

Mice infected with 10^5^ PFU of BJx109 and treated with either mAb 1A8 or with control IgG did not present clinical signs of disease or weight loss at any time-point (Fig. 4Ai), whereas BJx109-infected mice treated with mAb RB6-8C5 lost up to 10% of their original body weight, but recovered from infection (Fig. 4Bi). Mice infected with HKx31 and treated with either mAb 1A8 or mAb RB6-8C5 lost weight rapidly and all mice succumbed to disease at day 5 post-infection (Fig. 4Aii/4Bii), whereas all IgG-treated control animals survived infection. Compared to IgG-treated controls, treatment of PR8-infected mice with mAb RB6-8C5 was associated with a slight delay in weight loss in multiple experiments (Fig. 4Aiii) whereas PR8-infected mice treated with mAb 1A8 consistently succumbed to infection 1 day earlier than IgG-treated controls (Fig. 4Biii). Together, these data indicate that Ly6G^+^ neutrophils play a beneficial role in mice following challenge with influenza virus strains of moderate to high virulence. In contrast, specific depletion of neutrophils using mAb 1A8 did not alter the course of disease following infection with the avirulent BJx109 strain. Depletion of Gr-1^+^ cells (using mAb RB6-8C5) was, however, associated with significant weight loss in BJx109-infected mice.

### Effect of mAb 1A8 and mAb RB6-8C5 treatment on virus replication in the respiratory tract following intranasal infection

**Figure 5:**
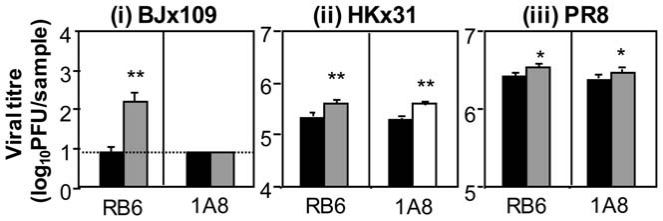
Virus replication in neutropenic and control mice following infection with BJx109, HKx31 and PR8. Groups of 5 B6 mice received i.n. and i.p. treatments with purified anti-Ly6G (1A8, striped bars) 24 hrs prior to virus infection and every second day thereafter. For comparison, virus-infected mice were also treated with anti-Gr-1 (RB6-8C5, grey bars) antibodies and control mice with rat IgG (IgG, black bars). Mice were infected with 10^5^ PFU of BJx109, HKx31 or PR8 and at day 5 post-infection mice were killed, lungs removed and titres of infectious virus were determined in clarified homogenates by standard plaque assay. Data represent mean viral titres titres ± 1 SD. The detection limit for the assay is indicated by the dotted line. Viral titres from treated mice (RB6-8C5 or 1A8) that were significantly higher than those from control (IgG-treated) animals are indicated by * = *p*<0.05, ** = *p*<0.01 (Student's *t*-test).

To examine the effect of neutrophils on virus replication in the airways, mice were infected with 10^5^ PFU of BJx109, HKx31 or PR8 and treated with mAb 1A8 prior to and during infection with each virus strain. Additional mice were treated with mAb RB6-8C5 while control mice received an equivalent treatment regime with purified rat IgG. Levels of infectious virus in the lung were determined at day 5 post-infection (Fig. 5). Treatment of BJx109-infected mice with mAb 1A8 did not alter viral load, whereas treatment with mAb RB6-8C5 led to enhanced virus replication and delayed clearance. Similar titres of infectious virus were also recovered from nasal homogenates of BJx109-infected mice treated with mAb 1A8 at this time (data not shown). Compared to IgG-treated controls, virus titres were somewhat elevated in the lungs of HKx31- and PR8-infected mice following treatment with either mAb 1A8 or mAb RB6-8C5. Thus, using mAb 1A8 we demonstrate that neutrophils can play an important role in limiting viral replication in the lungs of mice infected with HKx31 and PR8, but not BJx109. Depletion of Gr-1^+^ cells, but not Ly6G^high^ neutrophils, was associated with exacerbated disease (Fig. 4) and virus replication (Fig. 5) in BJx109-infected mice.

### Neutrophils play a protective role during severe influenza virus infections

**Figure 6:**
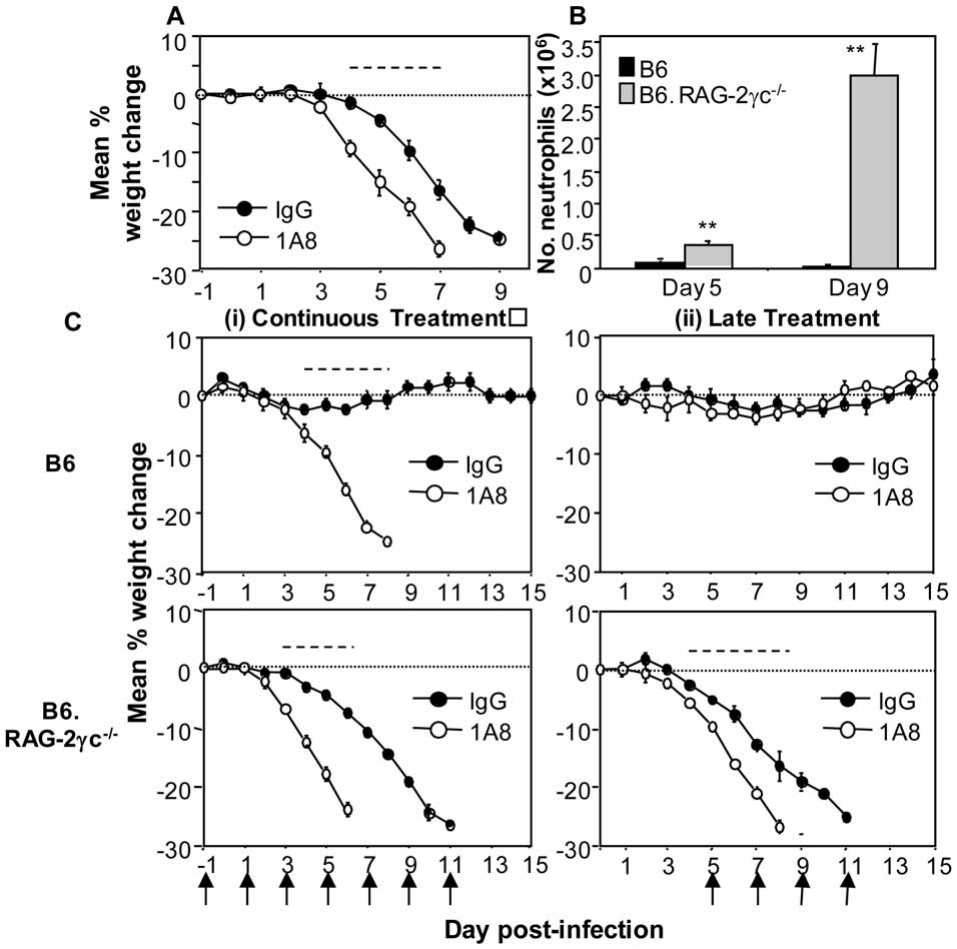
Neutrophils play a beneficial role during severe influenza infections of mice. (A) Groups of 5 B6 mice were treated with purified anti-Ly6G (1A8) or rat IgG (IgG) antibodies 24 hrs prior to virus infection and every second day thereafter. Data show weight loss for mice infected with 10^2^ PFU of PR8. (B) Groups of 5 B6 or B6.RAG-2γc^−/−^mice were infected with 10^2^ PFU of HKx31 and at day 5 and 9 post-infection, mice were killed and BAL performed. Numbers of BAL neutrophils from virus-infected mice are shown. Neutrophils were identified via differential counts and Diff Quick staining, as described in Material and Methods. * = neutrophil numbers from B6.RAG-2γc^−/−^ mice were significantly higher compared to numbers from B6 mice (*p*<0.01, one-way ANOVA). (C) Immunocompetent B6 mice (upper panels) or B6.RAG-2γc^−/−^mice (lower panels) were treated with purified anti-Ly6G antibodies (1A8) (i) at days −1, +1, +3, +5, +7, +9 and +11 (Continuous treatment, left panels), or (ii) at days +5, +7, +9 and +11 (Late treatment, right panels) relative to infection with 10^2^ PFU of HKx31 at day 0. Control mice received a similar treatment regime of rat IgG (IgG). Arrows indicate the days mice were treated with mAb 1A8 or control rat IgG. Mice were weighed daily and results expressed as the mean percent weight change of each group (± SEM), compared to original body weight. Animals that had lost ≥25% of their original body weight and/or presented with evidence of pneumonia were euthanized. Data shown are from one experiment and are representative of two independent experiments. Dashed lines indicate days on which the weight loss induced by treatment with (A/C) mAb 1A8 was statistically different to IgG-treated controls (*p*<0.05, Student's *t*-test).

Depletion of neutrophils was associated with a modest enhancement in disease severity and virus replication in mice infected with a high dose (10^5^ PFU) of HKx31 and PR8 but did not alter disease in BJx109-infected mice (Fig. 4, 5). This high inoculum dose was used to allow avirulent BJx109 to establish infection in the airways. Neutrophils have been proposed to contribute to lung pathology, rather than to protection, during PR8 infection of mice [9]. To investigate the role of neutrophils in more detail, mice were depleted of neutrophils using mAb 1A8 and infected with a low dose (10^2^ PFU) of the virulent PR8 strain (Fig. 6A). At this lower inoculum dose, neutropenic mice lost weight and succumbed to PR8 infection more rapidly than control animals further highlighting the beneficial role of neutrophils during severe influenza infections.

B6.RAG-2γc^−/−^ mice are deficient in B, T, NK and NKT cells [22] and therefore lack multiple components of innate and adaptive immunity. In the absence of multiple mediators of immunity, we hypothesized that the role of neutrophils during influenza virus infection could be studied more effectively. Compared to immunocompetent B6 animals, B6.RAG-2γc^−/−^ mice are highly susceptible to infection with a low dose of HKx31 (10^2^ PFU) and all mice reached the humane end-point for culling by day 10 post-infection (data not shown). We determined neutrophil numbers in the airways of HKx31-infected B6.RAG-2γc^−/−^ mice at day 5 and day 9 post-infection as these time-points correlated with development of mild and severe disease, respectively. Low numbers of neutrophils were recovered from the airways of B6 mice at either time point (Fig. 6B). In contrast, neutrophils numbers in BAL from B6.RAG-2γc^−/−^ mice were ∼5 and ∼200-fold higher than B6 mice at day 5 and 9, respectively, and by day 9∼80% of BAL cells were neutrophils. BAL neutrophils recovered from B6.RAG-2γc^−/−^ infected with 10^2^ PFU of HKx31 were 6–8 times higher than numbers from B6 mice infected with 10^5^ PFU of PR8 (Fig. 2B). Note that similar numbers of BAL neutrophils were recovered from the lungs of naïve B6 and B6.RAG-2γc^−/−^ mice (data not shown).

Excessive or dysregulated neutrophil recruitment has been proposed to contribute to inflammatory lung injury [23], [24] and to the pathology of influenza virus infections [9]. Given the massive neutrophil influx into the airways of B6.RAG-2γc^−/−^ mice, we hypothesized that under these conditions neutrophils may contribute to pathology and disease. Therefore, immunocompetent B6 mice and B6.RAG-2γc^−/−^ mice were infected with 10^2^ PFU of HKx31 and treated with mAb 1A8 or IgG at days (i) −1, +1, +3, +5, +7, +9 and +11 (‘continuous treatment’), or (ii) +5, +7, +9 and +11 relative to infection (‘late treatment’). Consistent with our previous findings [5], commencing treatment of HKx31-infected B6 mice with mAb 1A8 early after infection (‘continuous treatment’) led to rapid weight loss and death (Fig. 6Ci, upper panel) whereas ‘late treatment’ had no affect on the course of disease (Fig. 6Cii, upper panel). In contrast, both continuous (Fig. 6Bi, lower panel) and late treatments (Fig. 6Ci, upper panel) of B6.RAG-2γc^−/−^ mice with mAb 1A8 were associated with accelerated weight loss and disease in HKx31-infected mice. Together, these data indicate that the massive influx of neutrophils into the airways of HKx31-infected B6.RAG-2γc^−/−^ mice is not a major factor contributing to disease severity. Rather, the large numbers of airway neutrophils play a beneficial role in limiting disease severity and delaying mortality.

## Discussion

The current study contributes a number of important insights to our understanding regarding the role of neutrophils during influenza infection of mice. With one exception [5], previous studies have used mAb RB6-8C5 to address the role of neutrophils in the mouse model of influenza infection [3], [4], [8]. We have compared outcomes following treatment of mice with mAb 1A8 (anti-Ly6G, specific for neutrophils) or mAb RB6-8C5 (anti-Ly6G/C or anti-Gr-1, expressed by a range of additional leukocyte subsets) and report that depletion of Ly6G^+^ versus Gr-1^+^ cells in mice can be associated with different outcomes with respect to virus replication and disease. Moreover, our studies define the role of neutrophils following infection of mice with virus strains that are known to induce mild, intermediate or severe disease in mice. Finally, we have used virus-infected immunodeficient mice and mAb 1A8 to demonstrate that under conditions of excessive recruitment to the airways, neutrophils still play a protective rather than a pathological role.

Strain PR8 is highly adapted to growth in mouse lung and induces interstitial pneumonia [25], [26], similar to that observed in human cases of viral pneumonia. Multiple factors have been proposed to contribute to the virulence of PR8 including its high growth capacity, a function of the internal genes that led to its use in generation of high-yielding reassortant strains with vaccine potential [27]. BJx109, HKx31 and PR8 viruses share internal components derived from the PR8 strain but differ markedly in their ability to induce disease following intranasal inoculation of mice (Fig. 1), a finding attributed to the particular HA and/or NA glycoproteins of each virus strain. The differential ability of BJx109, HKx31 and PR8 to replicate and induce disease in mice (Fig. 1), recruit neutrophils to the upper and lower airways (Fig. 2) and the particular role that neutrophils play in controlling replication and disease (Fig. 4, 5) can be linked to the expression of distinct HA and NA glycoproteins by each of the virus strains used.

BJx109 replicates poorly and does not induce weight loss or disease in mice (Fig. 1) and the HA of BJx109 is highly glycosylated, rendering it sensitive to destruction by components of the innate immune system, including collectins [28], [29] and airway macrophages [30], [31]. In previous studies we have demonstrated that when specific components of innate immunity are depleted (e.g. airway macrophages) [16] or blocked (e.g. by intranasal treatment of mice with mannan to block lectin-mediated defenses) [32], BJx109 can replicate to high levels and induce severe disease and death in mice. In contrast, specific depletion of neutrophils via mAb 1A8 treatment did not enhance the virulence of BJx109 for B6 mice, suggesting that alternative immune defenses are sufficient to contain and clear infection. Intranasal infection of B6.RAG or B6.RAG-2γc^−/−^ mice with 10^5^ PFU of BJx109 does not induce weight loss or disease and BJx109-infected B6.RAG-2γc^−/−^ mice treated with mAb 1A8 did not show enhanced morbidity (MT and PR, unpublished observations). Our findings that mAb RB6-8C5, but not mAb 1A8, induced weight loss and increased virus replication in BJx109-infected mice would be consistent with recent findings that Gr-1 is upregulated on airway macrophages, as well as NK cells, NKT cells, cDC and pDC, during influenza virus infection [5]. Treatment of virus-infected mice with mAb RB6-8C5 is therefore likely to deplete a range of cells of the innate immune system, including airway macrophages.

Neutrophils have been implicated in lung pathology in a number of systems such as acute lung injury (ALI), acute respiratory distress syndrome (ARDS), septic shock and asthma (reviewed in [23], [24]). Previous studies demonstrated that treatment of PR8-infected mice with hyperimmune serum raised to neutrophil chemoattractant MIP-2 improved lung pathology without affecting virus replication and led to reduced morbidity and mortality [9], consistent with a role for neutrophils in driving the lung pathology associated with severe disease. High numbers of neutrophils were also recruited to the lungs of mice infected with pathogenic H1N1 and H5N1 influenza viruses [2], [3], yet depletion of Gr-1^+^ cells, including neutrophils (using mAb RB6-8C5 alone or in combination with clodronate-loaded liposomes to deplete airway macrophages) was associated with exacerbated growth and spread of a highly pathogenic recombinant virus expressing the HA and NA of the 1918 pandemic virus [3]. Despite progressive accumulation of neutrophils in the airways during infection with mouse-adapted PR8 (Fig. 2B), neutrophil-depleted mice succumbed more rapidly than controls (Fig. 4B). Infection of immunodeficient B6.RAG-2γc^−/−^ mice with a low dose (10^2^ PFU) of HKx31also induced massive neutrophil recruitment to the airways (Fig. 6A) yet neutrophil depletion during the later stages of infection (i.e. from day 5 onwards) did not ameliorate disease (Fig. 6B). Thus, data presented herein support the notion that neutrophils play a role in protection rather than contributing to the pathology associated with severe disease. Previous studies have used mAb RB6-8C5 to assess the role of neutrophils during severe influenza infections whereas our studies utilize Ly6G-specific mAb 1A8.

Incubation of human neutrophils with influenza virus has been associated with intracellular detection of virus-specific proteins [33], [34], [35], however infection was non-productive [33]. Furthermore, immunohistological localization of H5N1 viral proteins in the cytoplasm and nucleus of human neutrophils led to the proposal that neutrophils may act as a vehicle for viral transportation and dissemination during virulent influenza virus infections [34]. In the mouse model, previous studies have demonstrated that casein or glycogen-induced peritoneal neutrophils did not support productive virus replication [6]. Herein, we have compared the ability of virulent and avirulent virus strains to infect and replicate within purified bone marrow-derived neutrophils from naïve B6 mice. Consistent with previous studies [33], amplification of infectious progeny was not detected in cell supernatants 24 hrs after exposure to BJx109, HKx31 or PR8. Intracellular NP was not detected using flow cytometry (Fig. 3A) and levels of viral mRNA and vRNA were low compared to murine LA-4 epithelial cells (Fig. 3B). Of interest, levels of vRNA were markedly higher than mRNA, consistent with previous reports that neutrophils mediate phagocytic uptake of influenza virus [6], [36].

In conclusion, we have used antibody-mediated depletion of Ly6G^+^ cells to investigate the role of neutrophils during influenza virus infection of mice. We demonstrate that neutrophils play a role in limiting disease severity following infection of mice with stains of intermediate (HKx31; H3N2) and high virulence (PR8; H1N1) whereas they are not critical in controlling infection with avirulent BJx109. Previous studies have suggested that overwhelming or dysregulated neutrophil responses may contribute to lung injury in inflammatory conditions, however the large neutrophil influx associated with severe infections (i.e. PR8 in B6 mice or HKx31 in B6.RAG-2γc^−/−^ mice) provided a beneficial rather than a detrimental effect. In contrast to data obtained using mAb 1A8, BJx109-infected mice treated with mAb RB6-8C5 showed enhanced disease severity and virus replication in the airways. Together, these demonstrate that neutrophils can play differing roles in modulating influenza disease depending on the virulence of the particular strain examined. Moreover, our data confirm the limitations associated with use of mAb RB6-8C5 to define the role of neutrophils *in vivo* during inflammation and infection.

## Materials and Methods

### Mice and viruses

C57BL/6 (B6) mice and RAG-2 common gamma chain-deficient mice on a B6 background (B6. RAG-2γc^−/−^) were bred and housed in specific pathogen-free conditions at the Department of Microbiology and Immunology, University of Melbourne, Australia. Male 6–10 week old mice were used in all experiments. The influenza A virus strains used in this study were A/PR/8/34 (PR8, H1N1), as well as BJx109 (H3N2) and HKx31 (H3N2), which are high-yielding reassortants of PR8 that bear the surface glycoproteins of A/Beijing/353/89 (H3N2) and A/Aichi/2/68 (H3N2), respectively. Viruses were grown in 10-day embryonated hen's eggs by standard procedures and titrated on Madin-Darby canine kidney (MDCK) cells as described [37].

### Infection and treatment of mice

For total respiratory tract infection, mice were lightly anaesthetized with isoflurane and infected with 10^5^ PFU of BJx109, HKx31 or PR8 via the intranasal (i.n.) route in 50 µl of phosphate buffered saline (PBS). In some experiments, mice were inoculated with 10^2^ PFU of HKx31. Mice were weighed daily and assessed for visual signs of clinical disease including inactivity, ruffled fur, laboured respiration and huddling behaviour. Animals that lost ≥25% of their original body weight and/or displayed evidence of pneumonia were euthanized. All procedures were conducted in accordance with the University of Melbourne's Animal Experimentation Ethics guidelines and policies and approved by an institutional ethics committee (ID 0911208.1 approved by Biochemistry & Molecular Biology, Dental Science, Medicine (RMH), and Microbiology & Immunology committee). At various times after infection, mice were euthanized and the lungs and nasal tissues were removed, homogenised in PBS and clarified by centrifugation. Titres of infectious virus in tissue homogenates were determined by standard plaque assay on MDCK cells in the presence of trypsin.

For the depletion of Ly6G^high^ neutrophils or Gr-1^+^ leukocytes *in vivo*, mice were treated with purified anti-Gr-1 (Ly6G and Ly6C, RB6-8C5) or anti-Ly6G rat mAb (1A8, a gift from Prof. Thomas Malek, Dept. Microbiology and Immunology, University of Miami, Florida, USA), respectively. A combination of intraperitoneal (i.p.; 0.5 mg in 0.2 ml) and intranasal (i.n.; 0.2 mg in 0.05 ml) routes were used to obtain >90% depletion of neutrophils in the blood as well as >90% depletion in the airways of influenza virus-infected mice, as previously described [4], [5]. Mice were treated 24 hrs prior to infection and every 48 hrs thereafter, unless otherwise stated. Control animals received a similar dose of purified whole rat IgG (Jackson Laboratories, USA). Depletion of neutrophils in the blood (>95% compared to IgG-treated control mice) and airways (>80% compared to IgG-treated controls) was confirmed by differential leukocyte counts (data not shown).

### Recovery of leukocytes from mice

Bronchoalveolar lavage (BAL) cells and nasal tissue cells (nasal cavity and nasal turbinates) were obtained as described [4]. To obtain single cell suspensions, nasal tissues were finely minced with scissors, incubated for 30 minutes at 37°C with 2 mg/mL Collagenase A (Roche Diagnostics, Germany) and passed through a wire mesh. Samples were treated with Tris-NH_4_Cl (0.14 M NH_4_Cl in 17 mM Tris, adjusted to pH 7.2) to lyse erythrocytes and washed in RPMI 1640 medium supplemented with 10% FCS (RF_10_). Cell numbers and cell viability were assessed via trypan blue exclusion using a hemocytometer.

### Differential leukocyte counts and flow cytometry

BAL and blood samples were analyzed by differential leukocyte counts as described [4]. For flow cytometry analysis, single cell suspensions prepared from BAL, lung and nasal tissues were incubated on ice for 20 minutes with supernatants from hybridoma 2.4G2 to block Fc receptors and then stained with appropriate combinations of fluorescein isothiocyanate (FITC), phycoerythrin (PE), allophycocyanin (APC) or biotinylated monoclonal antibodies to Gr-1 (RB6-8C5) or CD45.2 (104). Viable cells were analysed by the addition of propidium iodide (PI; 10 µg/ml) to each sample and cells were analysed on a FACS Calibur flow cytometer. A minimum of 50,000 PI^−^ cells were collected. Leukocyte populations were sorted using a MoFlo cell sorter (DakoCytomation, Denmark).

### Detection of neutrophil chemoattractants in BAL supernatants

BAL supernatants from naïve and virus-infected mice were assessed for levels of MIP-1α (CCL3), MIP-2 (CXCL2) and KC (CXCL1) using ELISA kits (R&D Systems, USA) according to manufacturer's instructions. Chemoattractant concentrations were calculated from a standard curve and expressed as pg/mL.

### Purification of neutrophils from bone marrow

Mature neutrophils were purified from bone marrow of B6 mice by Dr. Ben Croker, Walter and Eliza Hall Institute of Medical Research, Melbourne, Australia, using a method adapted from Boxio *et al* [38]. Briefly, bone marrow cells were separated using a 3-layered gradient of 1.110 g/ml, 1.090 g/ml, and 1.083 g/ml Percoll PLUS (Amersham, Sweden). Percoll layers were prepared by diluting 100% Percoll (9 parts Percoll+1 part 10× HBSS, pH 7.1) with HBSS. After centrifugation (1500 *g*, 30 mins, no brake), neutrophils were collected from the 1.110 g/ml to 1.090 g/ml interface. Contaminants were further enriched via incubation with biotinylated anti-B220 and anti-Ter119 antibodies, followed by the addition of streptavidin-labelled Dynabeads (Invitrogen, USA). Cells were counted by trypan blue exclusion and purity was assessed to be >99% by differential counts of cytospin preparations stained with Diff-Quick (Amber Scientific, Australia).

### Infection of murine cells by influenza virus and detection of viral antigen using flow cytometry

For the detection of intracellular viral nucleoprotein (NP), 10^6^ purified mature bone-marrow neutrophils (prepared as described above) or LA-4 cells (murine airway epithelial cell line) were mock-infected with media alone or infected with 10^7^ PFU of influenza virus in suspension for 1 hr at 37°C. Cells were then washed and incubated for 8 hrs at 37°C. Cells were fixed with 80% (v/v) acetone and incubated with mAb MP3.10G2.IC7, specific for the nucleoprotein (NP) of type A influenza viruses (from WHO Collaborating Centre for Reference and Research on Influenza), followed by FITC-conjugated sheep anti-mouse Ig (Silenus, Australia) in the presence of 0.2% saponin. Levels of intracellular viral NP was determined using flow cytometry.

### Quantitative RT-PCR (qRT-PCR) for influenza virus genes

10^6^ purified mature bone-marrow neutrophils (prepared as described above) and LA-4 cells were incubated with 10^7^ PFU of influenza virus in suspension for 1 hr at 37°C. Cells were then washed and incubated for 8 hrs at 37°C. RNA was extracted from the cell pellet using the RNeasy Mini Kit (Qiagen, USA) according to manufacturer's instructions and stored at −70°C. Levels of viral RNA (vRNA) and viral messenger RNA (mRNA) of polymerase A subunit (PA) and matrix (M) genes were determined via qRT-PCR using TaqMan chemistry. Reactions contained 5 µl of RNA template, 1× TaqMan one-step RT-PCR master mix (Applied Biosystems, USA), 1× MultiScribe and RNase inhibitor mix (Applied Biosystems) and 0.5 µM of each primer and probe in a total reaction volume of 25 µl. For the detection of negative sense vRNA, the forward primer was added to the reaction mixture first, and the reverse transcriptase reaction was incubated at 50°C for 30 mins. The reverse primer was then added and quantitative PCR was performed using the following conditions; 94°C for 15 mins, followed by 40 cycles of denaturation/amplification (95°C for 30 secs, 60°C for 60 secs). For the detection of positive sense viral mRNA, the reverse primer was added to the reaction mixture first followed by the forward primer. A standard curve was created using serial dilutions of plasmids containing the target gene and the detection limit was 1 pg/ml, the lowest standard included in the assay. RNA content in test samples were expressed as pg/ml. The following primer and probe sequences were used: M; Forward: ATG CAA CGG TTC AAG TGA TCC T, Reverse: GTC AAG TGC AAG ATC CCA ATG AT, Probe: CAC TAT TGC CGC AAA T (VIC), and PA; Forward: ACA AGG CAT GCG AAC TGA CA, Reverse: TGG AGC CAC ATC TTC TCC AA, Probe: ATT CAA GCT GGA TAG AGC (VIC).

### Statistical analysis

For the comparison of two sets of values, a Student's *t* test (two-tailed, two-sample equal variance) was used. When comparing three or more sets of values, a one-way ANOVA test was applied followed by the Tukey post-test. A *p* value of ≤0.05 was considered statistically significant for all tests utilized.
